# Predicting high confidence ctDNA somatic variants with ensemble machine learning models

**DOI:** 10.1038/s41598-025-01326-2

**Published:** 2025-05-26

**Authors:** Rugare Maruzani, Liam Brierley, Andrea Jorgensen, Anna Fowler

**Affiliations:** 1 Department of Health Data Science, Institute of Population Health, Great Britain and Northern Ireland, University of Liverpool, Waterhouse Building, Block F, Brownlow Street, Liverpool, L69 3GF UK; 2 School Of Infection & Immunity, of Great Britain and Northern Ireland, Room 405, Sir Michael Stoker Building, Garscube Campus, 464 Bearsden Road, Glasgow, G61 1QH UK

**Keywords:** Cancer, Computational biology and bioinformatics

## Abstract

Circulating tumour DNA (ctDNA) is a minimally invasive cancer biomarker that can be used to inform treatment of cancer patients. The utility of ctDNA as a cancer biomarker depends on the ability to accurately detect somatic variants associated with cancer. Accurate somatic variant detection in circulating cell free DNA (cfDNA) NGS data requires filtering strategies to remove germline variants, and NGS artifacts. Rule-based variant filtering methods either remove a substantial number of true positive ctDNA variants along with false variant calls or retain an implausibly large number of total variants. Machine Learning (ML) enables identification of complex patterns which may improve ability to distinguish between real somatic ctDNA variants and false positive calls. We built two Random Forest (RF) models for predicting high confidence somatic ctDNA variants in low and high depth cfDNA NGS data. Low depth models were fitted and evaluated on whole exome sequencing (WES) cfDNA data at depths of approximately 10X while the high depth data was sequenced at approximately 500X. Both models utilise a set of 15 features from variants detected by bcftools, FreeBayes, LoFreq and Mutect2. High confidence ground truth sets were obtained from matched tissue biopsy samples. We benchmarked our models against rule-based filtering with a set of hard, medium, and soft thresholds. Precision-recall curves showed the high depth model outperformed rule-based filtering at all thresholds in Test Data (PR-AUC 0.71). Partial dependence plots showed membership in the COSMIC database, absence from the dbSNP common variants database, and increasing read depth increased mean probability of high confidence somatic variant prediction in both models. Our results demonstrate the utility of supervised ML models for filtering variants in cfDNA data.

**Supplementary Information:**

The online version contains supplementary material available at 10.1038/s41598-025-01326-2.

## Introduction

Circulating tumour DNA (ctDNA) is fragmented DNA released into the bloodstream by tumour cells. ctDNA has been shown to be a predictor of disease, relapse and prognosis for cancer patients^1–3^. ctDNA is minimally invasive to sample and has a short half-life, enabling near real-time monitoring for patients at risk of relapse^4^. Tumours can display intratumoral and spatial heterogeneity^5,6^ however as all tumour tissues release ctDNA, the entire genetic profile of a patient’s tumours can be analysed. This is in contrast to tissue biopsy where only genetic information at the biopsy site can be analysed^7^.

The utility of ctDNA as a cancer biomarker depends on the ability to accurately detect somatic variants associated with cancer. ctDNA can be in low abundance in the pool of total cell free DNA (cfDNA), particularly in the early stages of disease or when monitoring for recurrence. Additionally, the abundance of ctDNA fragments released from subclones will be even lower. Ultimately, it is difficult to distinguish between real low frequency ctDNA variants and NGS artifacts^8^.

Accurate somatic variant detection in cfDNA NGS data requires variant filtering methods. Accuracy of called variants is influenced by features including base quality and read depth. Base quality scores represent confidence in the called base by sequencing platforms. Read depth describes the total number of reads covering the variant site. Higher read depths and a higher number of reads supporting an alternate allele increase confidence a called variant is real. Rule-based filtering involves defining a set of thresholds for measures including read depth and base quality, and then discarding variants which fall below those thresholds. Rule-based filtering also often involves discarding variants reported in population databases including the 1000 Genomes Project^9^ and dbSNP^10^.

Another approach to the variant filtering problem is consensus, or ensemble variant calling.

Variant callers utilise a wide range of statistical methods and rely on differing assumptions about the input data to call variants. The result is that overlapping but different call sets are returned by different tools from the same input data. Variants commonly called by more than one caller have been shown to be more likely to be real somatic variants^11^. As such, ensemble variant calling aims to reduce the number of false positive calls by combining the output of multiple variant callers^12^. Naively, this may involve filtering out variants detected by a single caller^12^.

Both these filtering strategies have limitations. Depending on the thresholds specified, rule-based filtering can discard a large fraction of true positive variants or retain an implausibly large number of variants post-filtering. Benchmarking studies have shown variant callers have low concordance in variants detected from the same input data^13^. Naturally, ensemble variant call sets tend to have high precision, with a trade-off in low recall^14^. Machine Learning (ML) algorithms can enable identification of complex, non-linear patterns which may improve ability to distinguish between real somatic ctDNA variants and false positive calls.

Recently, ML tools for predicting somatic variants in cancer samples have been published^14–17^. However, few studies have focused on developing models for predicting somatic variants specifically in cfDNA NGS data. Jongbloed et al., (2023)^19^ present an ML model for detecting somatic variants in cfDNA data from breast cancer patients. The model, however, was fitted on, and developed for, targeted sequencing data. Depending on the size of the panel, targeted sequencing can return orders of magnitude fewer variants compared to Whole Exome Sequencing (WES), reducing the resources required to validate calls, and consequently, the need for specialist filtering tools.

Fitting supervised ML models requires a truth set to label positive examples. However, obtaining a truth set for predicting somatic variants in NGS data can be a challenge. This process may involve resource-intensive orthogonal sequencing or manual review of candidate variants. The use of multiple sequencing platforms and ultra-high sequencing depths often utilised for orthogonal validation^20^ can prohibitively increase the costs associated with obtaining a truth set. Given the labour-intensive nature of manually reviewing variants^15^, typically only a subset of candidate true positive variants are validated^16^, potentially impacting the performance of the fitted model.

One approach to obtaining a truth set is using common variants between matched tissue biopsy and the cfDNA sample. Tumour variants are much easier to detect in tissue than in ctDNA, and therefore the variants identified at low frequencies in ctDNA which are also confidently identified in the tissue sample are highly likely to be true positives. This method is less labour-intensive compared to manual curation and does not necessarily require independent sequencing technology. It therefore allows for a larger number of variants to be included in the truth set.

**Fig. 1:**
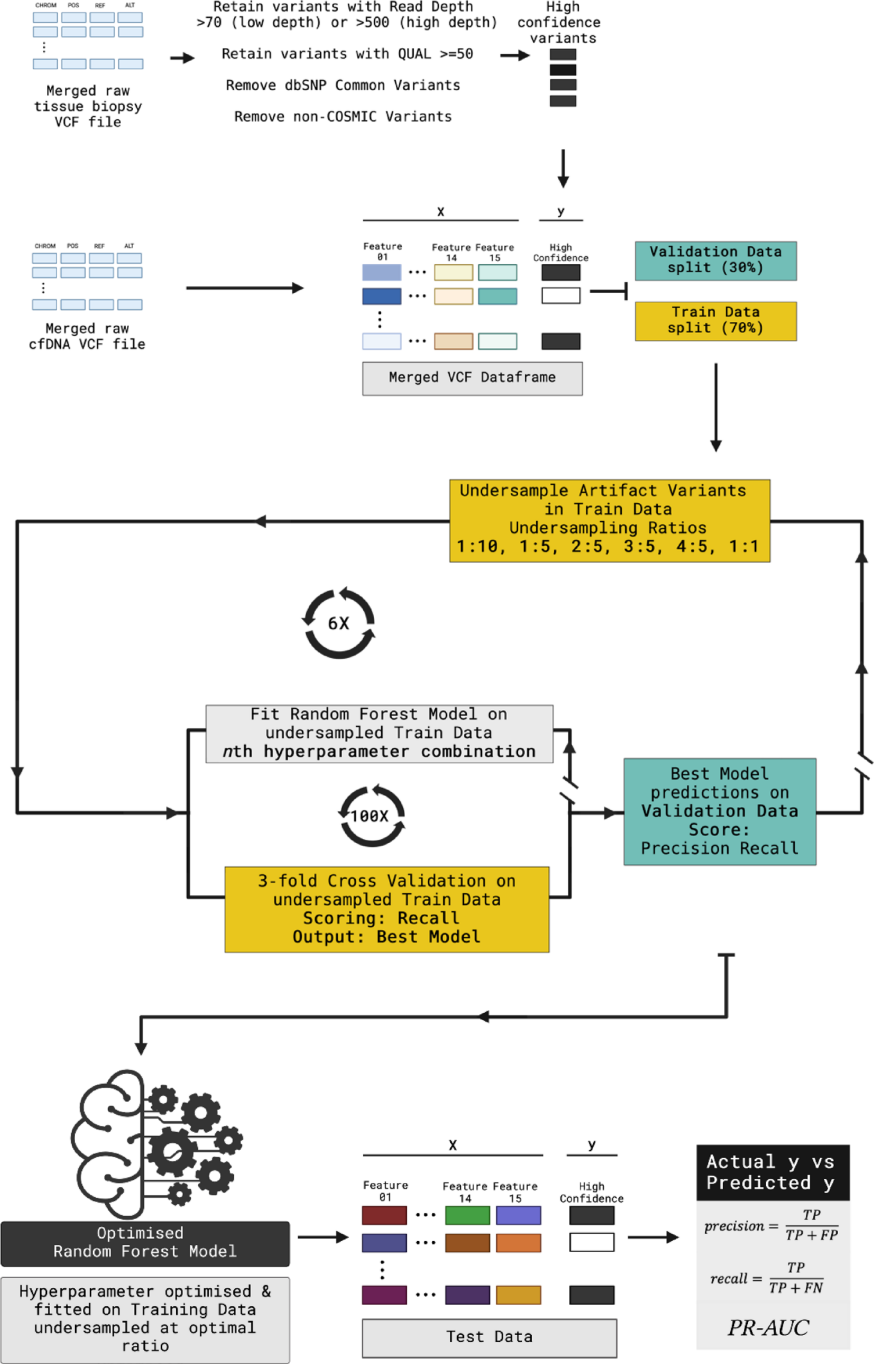
Workflow for training and evaluating ML models. In the outer loop, Training Data was randomly undersampled to each of six ratios. In the inner loop, hyperparameters were optimised for models fitted on undersampled data. The output best model was evaluated on Validation Data. Validation Data was not undersampled to reflect real data where ground truth is not known. The best performing model on Validation Data predictions was evaluated on previously unseen Test Data. Double brakes indicate loop break points at the end of iterations.

Here, we present two ML models for predicting high confidence somatic variants in low and high depth cfDNA WES datasets. The high confidence truth set was defined as variants detected in a matched tissue sample after stringent filtering. We extracted features from the output of 4 variant callers and predicted high confidence somatic variants with Random Forest models. Our models do not rely on matched normal samples; we utilise allele frequency and population databases to filter germline variants. The models were trained to predict single nucleotide variants (SNVs) only as they are the most common variant type in cancer. Figure 1 illustrates the workflow for model training and evaluation. Our code is available at https://github.com/rugare-m/somVar.

## Methods

### Datasets

We used a set of publicly available cfDNA WES samples to fit low and high depth ML models. The low depth model was trained and evaluated using samples from Dietz et al. (2016)^21^ ’s publication. This dataset included 6 cfDNA samples; each of the samples was sequenced with a matched tissue sample. The high depth model was trained and evaluated using samples from Li et al. (2022)^4^ and one sample from Butler et al. (2015)^22^, all sequenced with matched tissue samples. Table 1 shows the samples used to fit both models, including the accession numbers.

**Table 1:** Datasets used to train and test models. All samples are publicly available on the sequence read archive (SRA) with corresponding accession numbers.

cfDNA Accession	Matched Tissue	Reference	Cancer Type	Depth	Usage
SRR3401415	SRR3401405	Dietz et al., (2016)	Squamous cell carcinoma	Low	Train/Validation
SRR3401416	SRR3401406	Dietz et al., (2016)	Squamous cell carcinoma	Low	Train/Validation
SRR3401417	SRR3401411	Dietz et al., (2016)	Lung adenocarcinoma	Low	Train/Validation
SRR3401418	SRR3401412	Dietz et al., (2016)	lung adenocarcinoma	Low	Train/Validation
SRR3401407	SRR3401413	Dietz et al., (2016)	Lung adenocarcinoma	Low	Train/Validation
SRR3401408	SRR3401414	Dietz et al., (2016)	Squamous cell carcinoma	Low	Test
SRR12083523	SRR12083524	(Li et al., 2022)	Glioma	High	Train/Validation
SRR12083526	SRR12083527	(Li et al., 2022)	Glioma	High	Train/Validation
ERR855950	ERR855951	(Butler et al., 2015)	Sarcoma	High	Train/Validation
SRR12083529	SRR12083530	(Li et al., 2022)	Glioma	High	Test

### Data pre-processing

To generate BAM files, FASTQ files were mapped to GRCh38 using BWA-MEM2 v2.2.1^23^ at default parameters. Output SAM files were converted to BAM file format using Samtools v1.2^24^. Duplicate reads in BAM files were marked with GATK^25^ MarkDuplicates v4.3.0.0, before recalibrating base quality scores with GATK BaseRecalibrator v4.3.0.0 and GATK ApplyBQSR v4.3.0.0. The sequencing depth profiles of all processed BAM files was visualised by plotting a histogram of depth at every position in the GRCh38 exome.

### Variant calling pipeline

Four variant callers were used to detect variants; bcftools v1.16^26^, FreeBayes v1.3.6^27^, LoFreq v2.1.5^28^, Mutect2 v4.3.0.0^29^. All callers were run at default parameters. Output VCF files were decomposed to split multiallelic sites into individual records, and indels were removed. We discarded variants with allele frequencies between 0.4 and 0.6, and above 0.9 as these were assumed to be likely germline variants. VCF files were annotated with strand bias, mapping quality, base quality, fragment length, read position, allele frequency and number of reads supporting alleles using GATK v4.3.0.0 VariantAnnotator. SnpSift v4.3 t^30^ was used to annotate coding variants reported in COSMIC v98 and common variants in dbSNP v151. For each sample, VCF files from all callers were merged into a single VCF file using GATK MergeVcfs.

### Obtaining truth sets

We used a set of high confidence variants as true positive calls. These variants were defined as merged tissue variants with read depth greater than 70 for low depth data (or greater than 500 for high depth data), with Phred quality scores > = 50, not reported in the common variants dbSNP v151 VCF file and reported in the COSMIC v98 coding mutations VCF. To minimise the number of germline variants in the truth set, variants with previously described allele frequencies were discarded.

### Machine learning features

A set of 15 features per variant in the merged cfDNA VCF files were extracted into tabular data from data in the BAM, reference FASTA and VCF files. BAM level features included read depth, strand bias, median fragment length of reads supporting alternate allele, mapping quality, allele frequency, and median distance of variant from end of reads supporting alternate allele. The read depth feature was standardised by removing mean and scaling to unit variance using scikit-learn v1.4.1^31^. Reference FASTA features included GC percentage of reference sequence in a 20-nucleotide region flanking the variant (41 bases total), and a weighted homopolymer score calculated from the same region^32^. VCF features included a Boolean of presence of the variant in each of dbSNP and COSMIC databases, and bcftools, FreeBayes, LoFreq, and Mutect2 VCF files. Table 2 describes features used in the ML models. For the target, variants were labelled as high confidence somatic variants (HCSVs) if they were present in the truth VCF file and artefact variants (AVs) otherwise.

**Table 2:** Description of features used for training ML models.

Feature	Description
Read Depth	Total number of reads at variant locus calculated as reads supporting reference + reads supporting alternate
dbSNP	Is variant described in dbSNP v151 common variants database
COSMIC	Is variant described in the COSMIC v98 database
Strand Bias	Strand bias estimated using Fisher’s exact test, Phred-scaled p-value
Median Alt Fragment Length	Median fragment length of reads supporting alternate allele
Weighted Homopolymer Rate (WHR)	The sum of squares of the homopolymer lengths divided by the number of homopolymers in a 20-nucleotide region flanking the variant, including the variant. Homopolymers were defined as 4-mers and above.
GC Percentage	GC percentage in a 20-nucleotide region flanking the variant, including the variant
Mapping Quality	Median mapping quality of reads supporting alternate allele
Allele Frequency	Variant allele frequency calculated as (reads supporting alternate)/(reads supporting reference + reads supporting alternate)
bcftools	Is variant called by bcftools at default parameters
FreeBayes	Is variant called by FreeBayes at default parameters
LoFreq	Is variant called by LoFreq at default parameters
Mutect2	Is variant called by Mutect2 at default parameters
Distance from End of Read	Median distance of variant starts from ends of reads supporting alternate allele
Median alt base quality	Median base quality of bases supporting alternate allele

### Train, validation and test data

One test sample from each of the low and high depth datasets was chosen. This was kept independent of the training and validation pipeline. Variants from the remaining samples were allocated to training and validation sets, with a 70:30 training: validation split. The models were optimised using the training and validation datasets and then used to predict high confidence somatic variants in the test samples.

To select the production ML algorithm, we first evaluated a set of algorithms for performance with default hyperparameters in both low and high depth data, class balanced with a set of oversampling and undersampling methods (Supplementary Tables 1 and 2). Prioritising recall, Random Forest models, with randomly undersampled (RUS) training data returned the highest precision and recall scores in both low and high depth validation data. We therefore used Random Forest and RUS for developing the final models. We trained our models using the scikit-learn implementation of the Random Forest (RF) algorithm. The Random Forest algorithm fits decision trees on subsamples of input data and averages tree predictions to classify samples.

### Class balancing and hyperparameter optimisation loop

ML models fitted on imbalanced datasets perform poorly when predicting the minority class. These models tend to classify all new data as majority class, which is often the class of least interest^33^. Here, the ratio of true positive somatic variants to total variants per low depth sample was in the order of 1:1000. To mitigate against this, we treated the undersampling ratio as a parameter to be optimised in a nested loop that undersampled Artifact Variants (AVs) in Training Data to one of 6 ratios and optimised hyperparameters for models fitted on the undersampled Training Data (Fig. 1).

In the outer loop, Training Data was undersampled to each of 1:10, 1:5, 2:5, 3:5, 4:5 and 1:1 HCSV to AV ratios using the RandomUnderSampler (RUS) algorithm in the Python library imbalanced-learn v0.12.0^34^. RUS randomly downsamples AVs until the user-defined HCSVs to AV ratio is reached.

The inner loop optimised hyperparameters for RF models fitted on the undersampled Training Data using RandomizedSearchCV in scikit-learn. The random search was set to evaluate 100 different hyperparameter combinations using 3-fold cross validation. Three folds were selected to manage computation costs and variants were grouped per sample. Recall score was used for evaluating the best model. The best model from each of the 6 undersampling ratios was used to predict classes in Validation Data, and precision and recall scores were calculated. Validation Data was not undersampled to reflect real data where the ground truth is unknown.

Prioritising recall, the model with the highest precision and recall scores on Validation Data predictions was used to evaluate performance on the previously unseen Test Data. Class balancing ratios and hyperparameters were separately optimised for low and high depth models.

### High confidence somatic variant probability thresholds

The Random Forest algorithm predicts the probability of a variant being a HCSV. A default threshold of 0.5 is required to label variants as HCSV. Increasing the threshold increases the model precision score at the expense of recall. To reduce the false positive calls associated with high recall selected for in hyperparameter optimisation, we set the HCSV prediction threshold default to 0.75. Predictions in Test Data were with the threshold value set to 0.75.

### Evaluating models on test data

We produced confusion matrices on Test Data for the exported models to show the raw numbers of true positive (TP), true negative (TN), false positive (FP) and false negative (FN) predictions by the models. As ROC curves plot true positive rate (TPR) against false positive rate (FPR), they tend to be overly optimistic for models predicting on imbalance data. Poor models can have a low FPR resulting in misleading, high AUC scores. Precision-recall (PR) curves can provide a more accurate assessment of model performance on imbalanced datasets. However, PR curves may be unstable due to the low number of positive class samples. We ultimately opted to use precision-recall (PR) curves over receiver operator characteristics (ROC) to evaluate the models’ performance at different thresholds^14^ as we did not have low positive class numbers in absolute terms, instead having substantially more negative examples in both low and high depth datasets.

### Evaluating generalizability of random forest models to other cancer types

To evaluate the generalizability of RF models trained on one cancer type to other cancer types, we fitted RF models on one cancer type and evaluated on a different type for both low and high depth samples. For low depth samples, squamous cell carcinoma (SCC) was used to fit the RF model, and lung adenocarcinoma (LUAD) samples were used as Test Data. For high depth samples, the glioma samples were used for training, while the sarcoma sample was used as Test Data. Models were trained using previously optimised hyperparameters.

### Feature importance analysis

We assessed features’ contribution to model performance using permutation feature importance in scikit-learn. This method shuffles a feature in Validation Data *n* times and calculates the mean decrease of the model’s score on the permuted Validation Data. Here, each feature was shuffled 30 times and we used Average Precision (AP) as the scoring metric - AP summarises the precision-recall curve (Table 3). Next, we calculated partial dependence of the top three features from permutation feature importance analysis in the Validation Data. Partial dependence plots (PDPs) are calculated by varying a feature and observing resulting change in model-predicted marginal probability of HCSV, i.e. averaging out all other features.

**Table 3:** Description of evaluation metrics used to assess model performance.

	**Equation**	**Definition**
Precision	$\frac{{TP}}{{TP + FP}}$	The number of true positives (TP) over the number of true positives plus the number of false positives (FP)
Recall	$\frac{{TP}}{{TP + FN}}$	The number of true positives (TP) over the number of true positives plus the number of false negatives (FN).
Average Precision	$\mathop \sum \limits_n \left( {Rn - Rn - 1} \right)Pn$	The weighted mean of precisions (P) achieved at nth threshold (n), with the increase in recall (R) from the previous threshold used as the weight

### Benchmarking against rule-based filtering

We aimed to benchmark the RF models against rule-based filtering strategies using the SRR3401408 and SRR12083529 samples which were held out for testing. We used the RF models to predict high confidence variants on corresponding Test Data and plotted PR curves. Next, we performed rule-based filtering of the merged test sample cfDNA VCF files at three different thresholds; hard, medium, and soft, and plotted precision and recall on PR curves. Rule-based filtering at all thresholds included the previously described allele frequency filters to discard germline variants. Table 4 illustrates the thresholds applied at each filtering threshold.

**Table 4:** Rule-based filtering thresholds used to benchmark against RF models. Variants at or above thresholds were retained.

**Thresholds**	**Read depth low**	**Read depth high**	**QUAL**	**dbSNP ** **v151**	**COSMIC v98**
Hard	>20	>1000	>=50	0	1
Medium	>10	>750	>=40	0	1
Soft	>5	>500	>=20	0	1

## Results

### Sequencing depth profiles of matched tissue and test data samples

We applied a read depth filter in the matched tissue samples to obtain a HCSV truth set and for benchmarking models against rule-based filtering. The thresholds for read depth are dependent on the sequencing depths of the data. To inform selection of the appropriate read depth thresholds, we plotted histograms of read depths at every position in the human exome for all samples.

**Fig. 2:**
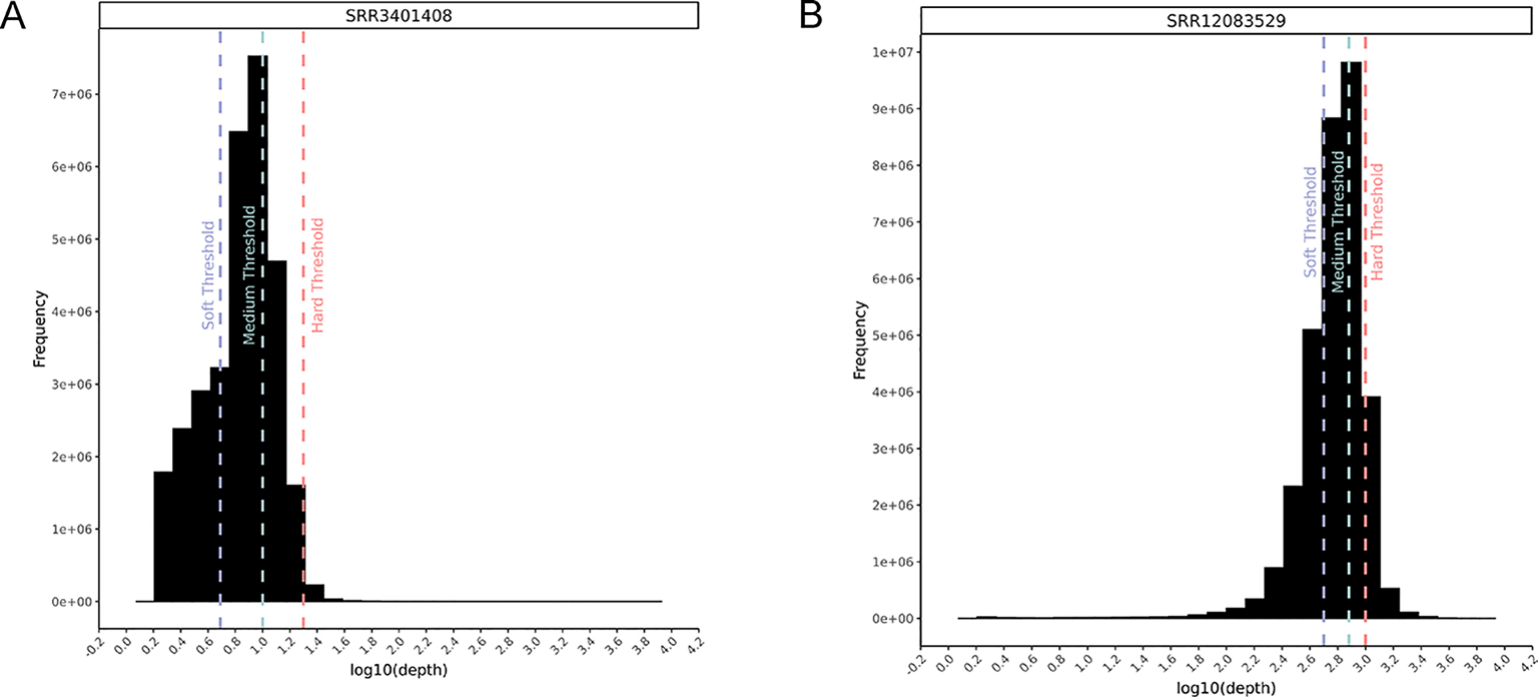
A-B: Sequencing depth histograms of low (**A**) high (**B**) depth test samples.

The low depth test sample showed a mode read depth of approximately 10X (Fig. 2A). 10X read depth was selected as the medium depth threshold, with 5X and 20X as the soft and hard thresholds respectively. In the high depth test sample, the mode was approximately 750X, which was selected as the medium depth threshold, with 500X and 1000X selected as soft and hard depth filter thresholds respectively (Fig. 2B).

Matched tissue samples showed read depth of approximately 50X and 200X in low and high depth data respectively. We defined high confidence variants as variants passing a stringent depth filter in matched tissue data. We therefore selected thresholds of 70X and 500X for low and high depth data truth sets respectively.

#### Variant counts

The low depth cfDNA samples returned a mean total of 311,766 SNVs, and 486 high confidence variants. The high depth samples returned a mean total of 710,437 SNVs, and 1,509 high confidence variants. ERR855950 returned a substantially higher total number of variants compared to all other high depth samples with 2,013,269 SNVs (Table 5).

**Table 5:** Total number and high confidence variants returned by from low and high depth datasets.

**cfDNA Accession**	**Total Number of Variants**	**High Confidence Variants**	**Depth**
SRR3401415	298,225	376	Low
SRR3401416	318,772	446	Low
SRR3401417	261,362	286	Low
SRR3401418	411,688	602	Low
SRR3401407	358,119	838	Low
SRR3401408	222,430	369	Low
SRR12083523	257,374	1,446	High
SRR12083526	309,852	1,592	High
ERR855950	2,013,269	1,183	High
SRR12083529	261,256	1,817	High

#### Hyperparameter and class balancing optimisation

We fitted two Random Forest models for predicting high confidence somatic ctDNA variants. We optimised the hyperparameters of each model using a random search and 3-fold cross validation.

Low depth models fitted on data undersampled to ratios of 2:5, 3:5, and 4:5 all returned the greatest performance, with 1.00 and 0.07 recall and precision scores. To select the best model from this set of 3, we compared the PR areas under curve (PR-AUCs) on Validation Data predictions. The PR-AUC metric ranges from 0 to 1, with a perfect classifier returning a PR-AUC 1 while a model with no predictive power returns a PR-AUC of 0. Here, the model fitted on Training Data undersampled to 2:5 returned the highest score of 0.43. The 3:5 and 4:5 models returned PR-AUCs of 0.31 and 0.28 respectively. The model fitted on Training Data undersampled to 2:5 was selected as the final low depth model. Undersampling the low depth Training Data to a 2:5 ratio reduced AVs from 1,151,933 to 4,457.

The high depth model fitted on Training Data undersampled to 1:5 ratio returned the highest precision and recall scores; 0.15 and 1.00 respectively (Table 6). Undersampling high depth Training Data to 1:5 ratio reduced AVs variants from 1,803,351 to 14,975.

**Table 6:** Precision and Recall scores of low and high depth models predicting classes on Validation Data.

		**Low depth**	**High depth **
1:10	Precision	0.09	0.20
	Recall	0.94	0.99
1:5	Precision	0.07	0.15
	Recall	0.99	1.00
2:5	Precision	0.07	0.13
	Recall	1.00	1.00
3:5	Precision	0.07	0.14
	Recall	1.00	1.00
4:5	Precision	0.07	0.14
	Recall	1.00	0.99
1:1	Precision	0.03	0.13
	Recall	0.96	0.99

#### High depth random forest model outperformed rule-based filtering

**Fig. 3:**
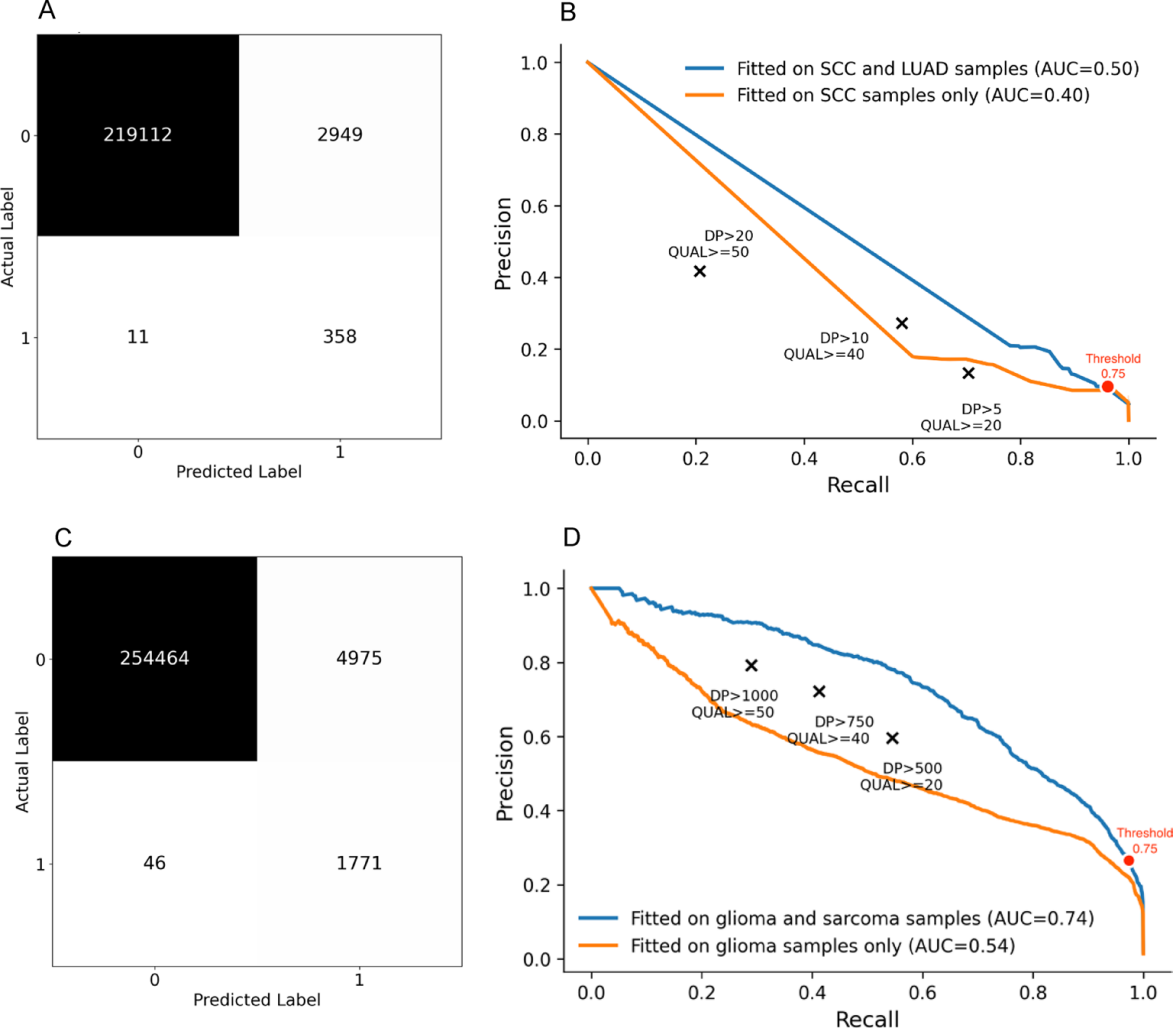
**A) **Confusion matrix for low depth model predictions on SRR3401408. The model was fitted on both SCC and LUAD samples.** B) **Precision Recall curves of low depth models. The SCC and LUAD model Test Data was SCC sample SRR3401408. The SCC only model Test Data was LUAD samples SRR3401407 SRR3401417, and SRR3401418.**C) **Confusion matrix for high depth model predictions on SRR12083529. Model was fitted on both glioma and sarcoma samples.** D) **Precision Recall curves of high depth models. The glioma and sarcoma model Test Data was sarcoma sample SRR12083529. Glioma only model Test Data was sarcoma sample ERR855950.

We evaluated the performance of both models using PR-AUC, and confusion matrices on Test Data predictions. Low and high depth model PR-AUCs applied to these unseen data were 0.50 and 0.71 respectively (Fig. 3). The low depth model fitted on SCC samples and tested on LUAD samples returned a PR-AUC of 0.40, while the high depth model fitted on glioma samples and tested on the sarcoma sample returned a PR-AUC of 0.54.

The high depth model outperformed soft, medium, and hard rule-based filtering thresholds at predicting HCSV in respective Test Data (Fig. 3B). At a 0.75 HCSV probability threshold, the low depth model returned a recall of 0.97 and precision of 0.11; and the high depth model returned a recall of 0.97 and precision of 0.26 in respective Test Data predictions. Figure 3 illustrates the confusion matrices and PR-AUCs for low and high depth model predictions.

**Fig. 4:**
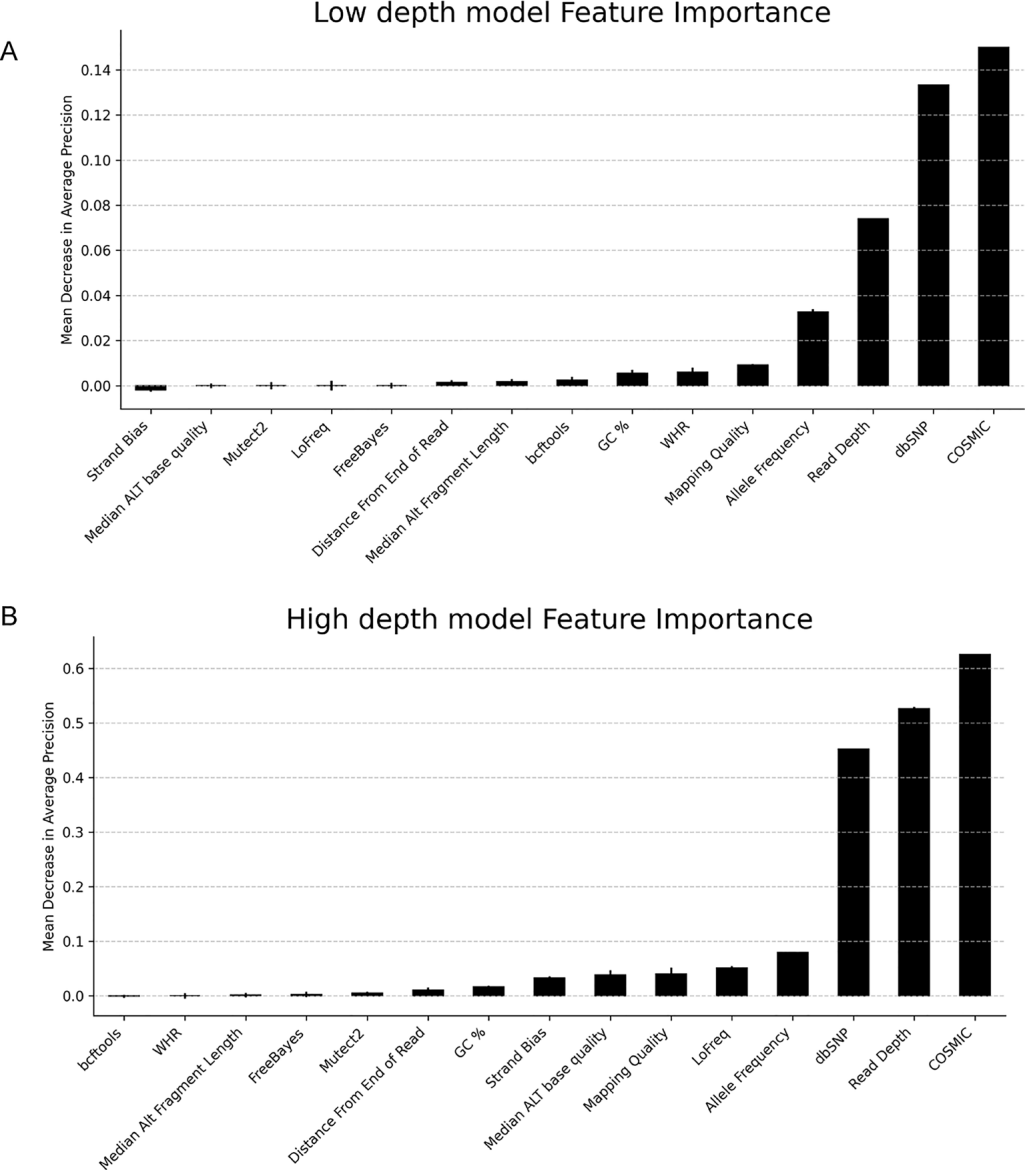
Permutation feature importance rankings for low (**A**) and high (**B**) depth models calculated in Validation Data. Error bars indicate the standard deviation across 30 permutations per feature.

#### Read depth, COSMIC and DbSNP membership are key features for ML models

We used permutation feature importance to assess the contribution of features in predicting HCSV (Fig. 4). Presence in COSMIC and dbSNP, and Read Depth were the top 3 features contributing to both models’ performance. Random permutation of COSMIC, dbSNP and Read Depth features resulted in a mean decrease in AP of 0.15, 0.13 and 0.07 respectively in low depth data predictions. In high depth data, the mean decrease in AP after permuting COSMIC, Read Depth and dbSNP were 0.63, 0.53 and 0.45 respectively. Permuting Median Alternate Fragment Length notably had a relatively low impact on AP in both model predictions.

**Fig. 5:**
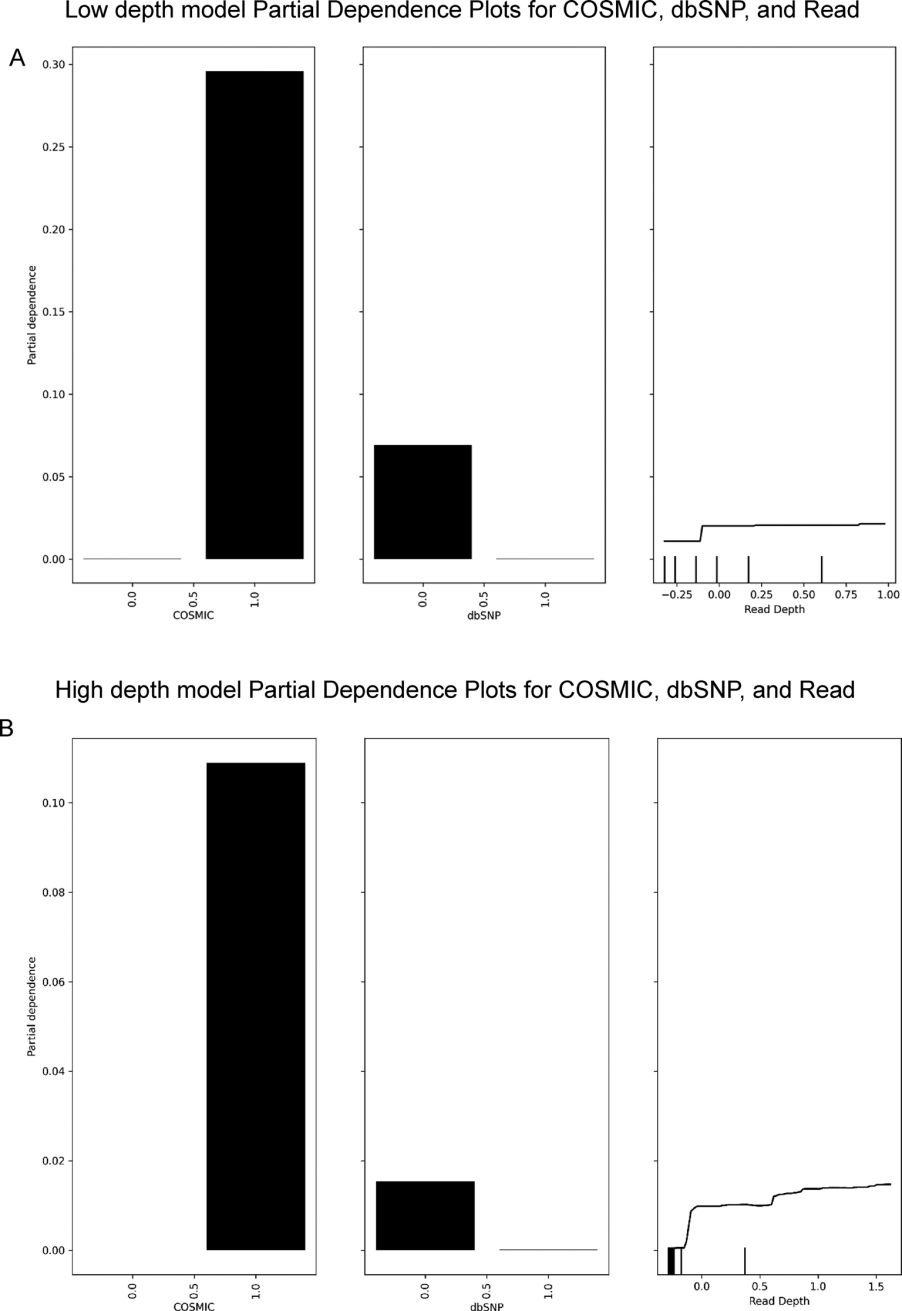
PDPs of the top 3 features in low depth (**A**) and high depth (**B**) models.

The PDPs showed variant presence in the COSMIC database results in the largest increase in mean probability of HCSV predictions for both models (Fig. 5). Absence in dbSNP also increased mean probability of HCSV predictions in both models. Increase in read depth increased mean probability of HCSV predictions, however the increase is small and plateaus, particularly in the low depth model.

## Discussion

Variant filtering is an important step in detecting somatic mutations in WES cfDNA NGS data. The aim is to filter out false positive calls without discarding too many real variants. Variant filtering in cfDNA data is complicated by the fact that ctDNA is typically in low abundance relative to healthy cfDNA. Ultimately true positive ctDNA variants occur at low allele frequencies that are difficult to distinguish from sequencing and PCR artifacts. We developed two ML models for predicting high confidence somatic ctDNA variants in low and high depth data. Our models were trained on WES samples with high confidence labels acquired from matched tissue samples. At a probability threshold of 0.75, both models correctly classified over 97% of HCSVs.

Hyperparameter optimisation for both models used recall as the scoring metric. While a balance of precision and recall is important, we determined recall is the more important metric in variant filtering of liquid biopsy NGS data. False positive variants can be removed in downstream orthogonal validation^15,35^; however, there is no way to rectify potentially actionable variants being discarded. Variants detected in a clinical context by any tool will likely require confirmation with Sanger sequencing for the foreseeable future^36^. The goal is to reduce the number of variants requiring confirmation, in turn reducing the resources required for validating variants, while retaining as many true positive variants as possible. The balance between precision and recall, however, can often be dependent on application of the tool. We therefore implemented a HCSV probability threshold parameter in our models to allow users to set the trade-off between precision and recall.

We generated partial dependence plots for the top-ranking features for each of our models. PDPs provide insight into how ML models make predictions. In both models, presence in COSMIC increased the mean probability of HCSV predictions more than any other feature. This was as expected given that the COSMIC database is a well-curated catalogue of cancer-associated variants, and the ground truth contains COSMIC variants only. Absence from dbSNP also increased probability of HCSV predictions in both models. This was expected as variants described in dbSNP are unlikely to be cancer associated somatic variants.

Read depth, mapping quality and base quality are routinely used features in ML models for predicting variants in NGS data^14,17,18,37^. In addition to these features, we included median fragment length of reads supporting the alternate allele in our models for predicting high confidence ctDNA somatic variants. Other researchers have previously demonstrated ctDNA fragments tend to be shorter than healthy cell free DNA^38,39^. True somatic ctDNA variants, therefore, are more likely to originate from shorter fragments compared to germline variants, and artefacts in normal cfDNA fragments. Permutation feature importance however demonstrated this feature had minimal effect on AP when permuted suggesting low predictive power in both models.

To evaluate generalisability of both our Random Forest models, we additionally fitted models on SCC samples and tested on LUAD samples for low depth data; and fitted on glioma samples and tested on the sarcoma sample for high depth data. The PR-AUCs show these models returned lower AUC scores compared to models tested on the same cancer type as used for training. The low generalisability of RF models may be explained by differences in mutational profiles between cancer types. Several studies have shown the differences in cancer mutation profiles including number of mutations per megabase and frequencies of transition and transversion mutations^42,43^. Additionally, the difference in fraction of high confidence somatic variants between the training data and the test data may contribute to lower AUC scores. In high depth data for example, high confidence variants in the glioma samples made up approximately 0.5% of total variants, compared to approximately 0.06% in the sarcoma sample. These results highlight the importance of predicting high confidence variants with ML models in data that matches the training data.

One of the known sources of sequencing errors in Illumina platforms is base calling in homopolymer regions. After a run of the same base, Illumina platforms may erroneously substitute the first base after the homopolymer with the homopolymer base. Stoler and Nekrutenko, (2021)^40^ found up to 5.3% of sequencing errors can be attributed to this effect. Repetitive regions are also associated with low mapping quality and base qualities^41^. We included the WHR feature in our models to filter out errors associated with repetitive sequences in the genome. Feature importance analysis, however, suggests WHR also has little predictive power.

While both models showed good performance, our model training and evaluation pipeline had some limitations. Filtering out germline variants in somatic variant calling pipelines can be a challenge without a matched normal sample^44^. Here, we attempted to remove germline variants by discarding variants reported in dbSNP and variants with an allele frequency of between 0.4 and 0.6, and above 0.9. Using population frequency databases to filter germline variants is a well-established approach [45, 46]. However, it is possible we discard some real HCSVs with the allele frequency filter.

Another limitation in our approach was assembly of the truth set. Commonly detected variants in cfDNA and matched tissue samples after stringent filtering were labelled true positive high confidence variants. The matched tissue biopsy for each sample only represents a fraction of the tumour tissues releasing cfDNA, therefore the ground truth sets were necessarily incomplete. A possible consequence of this is that the false positive rate in our models is inflated if the models are predicting real HCSVs that are absent from the tissue biopsy sample, and so the truth set.

Some approaches may be investigated to mitigate this limitation of our approach. The truth set may be derived from multiple tissue biopsies from the same patient. This approach would increase the probability of capturing all somatic variants compared to a single biopsy approach. The challenge of this approach is the increased sequencing costs and disadvantages associated with collecting biopsies from patients. Orthogonal validation of predicted variants may also be employed to more accurately assess model performance. However, the large number of high confidence variants returned from WES experiments may render orthogonal validation of all variants unfeasible as previously discussed.

To balance the classes in our training data, we randomly undersampled the majority class. Other approaches for training on imbalanced datasets are available and may be evaluated in future work. Weighted Random Forest (WRF), for example, can assign a weight to each class, with larger weights assigned to the minority class examples, giving higher penalty for misclassification. Future work may focus on benchmarking WRF compared resampling for datasets with extreme class imbalance.

## Conclusions

In this study we built two Random Forest models for predicting high confidence somatic variants in cfDNA from cancer patients. Filtering variants based on a set of thresholds can retain too many false positive calls or discard too many true positive calls. Here, we show ML methods have potential to outperform rule-based variant filtering. Our data also demonstrate the importance of test datasets matching training data. Finally, there is a need for high confidence truth datasets for cancer cfDNA samples to enable training of more accurate ML models. Here, we obtained truth sets from matched tissue variants, an approach that results in an incomplete ground truth set.

## Electronic supplementary material

Below is the link to the electronic supplementary material.

Supplementary Material 1

## Data Availability

Sequence data that support the findings of this study have been deposited in the European Nucleotide Archive with the primary accession code PRJNA318450, SRR12083523, SRR12083524, SRR12083526, SRR12083527, ERR855950, ERR855951 SRR12083529, SRR12083530.
