# Supplementary material for: Predicting high confidence ctDNA somatic variants with ensemble machine learning models
Source: Sci Rep. 2025 May 26;15:18384. doi: 10.1038/s41598-025-01326-2 (PMC12106698; doi:10.1038/s41598-025-01326-2)
Supplement: Supplementary file 1 — Supplementary Material 1 [file 41598_2025_1326_MOESM1_ESM.docx]

|  | 1:10 | | 1:5 | | 2:5 | | 3:5 | | 4:5 | | 1:1 | |
| --- | --- | --- | --- | --- | --- | --- | --- | --- | --- | --- | --- | --- |
|  | **Pr.** | **Re.** | **Pr.** | **Re.** | **Pr.** | **Re.** | **Pr.** | **Re.** | **Pr.** | **Re.** | **Pr.** | **Re.** |
| SMOTE | 0.08 | 0.73 | 0.06 | 0.83 | 0.04 | 0.89 | 0.04 | 0.91 | 0.04 | 0.93 | 0.02 | 0.94 |
| NearMiss | 0.03 | 0.97 | 0.02 | 0.98 | 0.02 | 1.00 | 0.02 | 1.00 | 0.02 | 1.00 | 0.02 | 1.00 |
| RUS | 0.08 | 0.72 | 0.06 | 0.83 | 0.04 | 0.89 | 0.03 | 0.93 | 0.03 | 0.93 | 0.02 | 0.92 |
| ROS | 0.08 | 0.73 | 0.06 | 0.84 | 0.04 | 0.91 | 0.04 | 0.92 | 0.03 | 0.94 | 0.02 | 0.90 |

A: LR

|  | 1:10 | | 1:5 | | 2:5 | | 3:5 | | 4:5 | | 1:1 | |
| --- | --- | --- | --- | --- | --- | --- | --- | --- | --- | --- | --- | --- |
|  | **Pr.** | **Re.** | **Pr.** | **Re.** | **Pr.** | **Re.** | **Pr.** | **Re.** | **Pr.** | **Re.** | **Pr.** | **Re.** |
| SMOTE | 0.50 | 0.81 | 0.48 | 0.82 | 0.47 | 0.82 | 0.47 | 0.82 | 0.47 | 0.82 | 0.46 | 0.81 |
| NearMiss | 0.10 | 0.99 | 0.10 | 0.99 | 0.10 | 0.99 | 0.10 | 1.00 | 0.10 | 1.00 | 0.10 | 1.00 |
| RUS | 0.22 | 0.98 | 0.18 | 0.99 | **0.15** | **1.00** | 0.14 | 1.00 | 0.13 | 1.00 | 0.13 | 1.00 |
| ROS | 0.76 | 0.59 | 0.76 | 0.60 | 0.75 | 0.61 | 0.75 | 0.60 | 0.76 | 0.61 | 0.76 | 0.61 |

B: RF

|  | 1:10 | | 1:5 | | 2:5 | | 3:5 | | 4:5 | | 1:1 | |
| --- | --- | --- | --- | --- | --- | --- | --- | --- | --- | --- | --- | --- |
|  | **Pr.** | **Re.** | **Pr.** | **Re.** | **Pr.** | **Re.** | **Pr.** | **Re.** | **Pr.** | **Re.** | **Pr.** | **Re.** |
| SMOTE | 0.53 | 0.78 | 0.48 | 0.80 | 0.47 | 0.82 | 0.45 | 0.82 | 0.43 | 0.84 | 0.43 | 0.85 |
| NearMiss | 0.10 | 0.98 | 0.10 | 0.99 | 0.10 | 0.99 | 0.09 | 0.99 | 0.09 | 0.99 | 0.09 | 0.99 |
| RUS | 0.21 | 0.98 | 0.18 | 0.99 | 0.16 | 0.99 | 0.14 | 1.00 | 0.13 | 1.00 | 0.12 | 1.00 |
| ROS | 0.38 | 0.90 | 0.36 | 0.90 | 0.34 | 0.92 | 0.33 | 0.92 | 0.32 | 0.91 | 0.32 | 0.92 |

C: XGB

|  | 1:10 | | 1:5 | | 2:5 | | 3:5 | | 4:5 | | 1:1 | |
| --- | --- | --- | --- | --- | --- | --- | --- | --- | --- | --- | --- | --- |
|  | **Pr.** | **Re.** | **Pr.** | **Re.** | **Pr.** | **Re.** | **Pr.** | **Re.** | **Pr.** | **Re.** | **Pr.** | **Re.** |
| SMOTE | 0.29 | 0.89 | 0.26 | 0.92 | 0.23 | 0.94 | 0.22 | 0.95 | 0.21 | 0.96 | 0.20 | 0.96 |
| NearMiss | 0.32 | 0.41 | 0.09 | 0.46 | 0.00 | 0.56 | 0.00 | 0.61 | 0.00 | 0.66 | 0.00 | 0.74 |
| RUS | 0.17 | 0.98 | 0.14 | 0.99 | 0.13 | 0.99 | 0.09 | 1.00 | 0.08 | 1.00 | 0.07 | 1.00 |
| ROS | 0.27 | 0.91 | 0.24 | 0.93 | 0.22 | 0.94 | 0.21 | 0.95 | 0.20 | 0.96 | 0.19 | 0.96 |

D: SVM

Supplementary Table 1: Precision and recall scores Logistic Regression (LR) (A), Random Forest (B), XGBoost (XGB) (C) and Support Vector Machine (SVM) (D) on Validation Data with default hyperparameters, with a threshold of 0.5. Models were fitted using high depth data. Green highlights indicate overall highest scoring model. Yellow highlights indicate highest scoring models in all other algorithms. Ratios indicate the target ratio of high confidence variants to artefacts after class-balancing.

|  | 1:10 | | 1:5 | | 2:5 | | 3:5 | | 4:5 | | 1:1 | |
| --- | --- | --- | --- | --- | --- | --- | --- | --- | --- | --- | --- | --- |
|  | **Pr.** | **Re.** | **Pr.** | **Re.** | **Pr.** | **Re.** | **Pr.** | **Re.** | **Pr.** | **Re.** | **Pr.** | **Re.** |
| SMOTE | 0.01 | 0.13 | 0.01 | 0.21 | 0.00 | 0.00 | 0.00 | 0.00 | 0.00 | 0.00 | 0.00 | 0.98 |
| NearMiss | 0.02 | 0.81 | 0.02 | 0.89 | 0.01 | 0.01 | 0.01 | 0.96 | 0.01 | 0.97 | 0.01 | 0.92 |
| RUS | 0.01 | 0.14 | 0.01 | 0.24 | 0.00 | 0.00 | 0.05 | 1.00 | 0.00 | 0.98 | 0.04 | 1.00 |
| ROS | 0.02 | 0.28 | 0.00 | 0.02 | 0.00 | 0.00 | 0.00 | 0.98 | 0.00 | 0.98 | 0.00 | 0.98 |

A: LR

|  | 1:10 | | 1:5 | | 2:5 | | 3:5 | | 4:5 | | 1:1 | |
| --- | --- | --- | --- | --- | --- | --- | --- | --- | --- | --- | --- | --- |
|  | **Pr.** | **Re.** | **Pr.** | **Re.** | **Pr.** | **Re.** | **Pr.** | **Re.** | **Pr.** | **Re.** | **Pr.** | **Re.** |
| SMOTE | 0.34 | 0.50 | 0.34 | 0.52 | 0.33 | 0.52 | 0.34 | 0.52 | 0.33 | 0.52 | 0.33 | 0.53 |
| NearMiss | 0.04 | 0.99 | 0.04 | 1.00 | 0.02 | 1.00 | 0.04 | 1.00 | 0.03 | 1.00 | 0.02 | 1.00 |
| RUS | 0.12 | 0.96 | 0.10 | 0.98 | 0.08 | 0.99 | 0.08 | 0.99 | 0.08 | 0.99 | **0.07** | **1.00** |
| ROS | 0.64 | 0.26 | 0.63 | 0.26 | 0.60 | 0.25 | 0.61 | 0.25 | 0.61 | 0.25 | 0.61 | 0.25 |

B: RF

|  | 1:10 | | 1:5 | | 2:5 | | 3:5 | | 4:5 | | 1:1 | |
| --- | --- | --- | --- | --- | --- | --- | --- | --- | --- | --- | --- | --- |
|  | **Pr.** | **Re.** | **Pr.** | **Re.** | **Pr.** | **Re.** | **Pr.** | **Re.** | **Pr.** | **Re.** | **Pr.** | **Re.** |
| SMOTE | 0.41 | 0.40 | 0.37 | 0.40 | 0.36 | 0.43 | 0.33 | 0.46 | 0.34 | 0.42 | 0.34 | 0.49 |
| NearMiss | 0.04 | 0.98 | 0.03 | 0.99 | 0.03 | 1.00 | 0.03 | 1.00 | 0.03 | 1.00 | 0.03 | 1.00 |
| RUS | 0.11 | 0.95 | 0.10 | 0.98 | 0.08 | 0.98 | 0.08 | 0.99 | 0.08 | 0.99 | 0.07 | 0.99 |
| ROS | 0.20 | 0.73 | 0.18 | 0.78 | 0.17 | 0.79 | 0.16 | 0.80 | 0.16 | 0.82 | 0.16 | 0.80 |

C: XGB

|  | 1:10 | | 1:5 | | | 2:5 | | 3:5 | | 4:5 | | 1:1 | |
| --- | --- | --- | --- | --- | --- | --- | --- | --- | --- | --- | --- | --- | --- |
|  | **Pr.** | **Re.** | **Pr.** | **Re.** | | **Pr.** | **Re.** | **Pr.** | **Re.** | **Pr.** | **Re.** | **Pr.** | **Re.** |
| SMOTE | 0.14 | 0.71 | 0.12 | | 0.76 | 0.11 | 0.81 | 0.1 | 0.83 | 0.1 | 0.85 | 0.1 | 0.86 |
| NearMiss | 0.00 | 0.21 | 0.00 | | 0.58 | 0.00 | 0.78 | 0.00 | 0.81 | 0.00 | 0.82 | 0.00 | 0.83 |
| RUS | 0.09 | 0.97 | 0.06 | | 1.00 | 0.06 | 1.00 | 0.06 | 1.00 | 0.06 | 1.00 | 0.06 | 1.00 |
| ROS | 0.13 | 0.74 | 0.11 | | 0.79 | 0.1 | 0.83 | 0.1 | 0.85 | 0.09 | 0.87 | 0.09 | 0.88 |

D: SVM

Supplementary Table 2: Precision and recall scores LR (A), RF (B), XGB (C) and SVM (D) on Validation Data with default hyperparameters, with a threshold of 0.5. Models were fitted using low depth data. Green highlights indicate overall highest scoring model. Yellow highlights indicate highest scoring models in all other algorithms. Ratios indicate the target ratio of high confidence variants to artefacts after class-balancing.
